# Taste-Masked Flucloxacillin Powder Part 2: Formulation Optimisation Using the Mixture Design Approach and Storage Stability

**DOI:** 10.3390/ph16081179

**Published:** 2023-08-18

**Authors:** Okhee Yoo, Sam Salman, Britta S. von Ungern-Sternberg, Lee Yong Lim

**Affiliations:** 1 Pharmacy, School of Allied Health, University of Western Australia, Perth, WA 6009, Australia; okhee.yoo@uwa.edu.au; 2 Clinical Pharmacology and Toxicology Unit, PathWest Laboratory Medicine, Perth, WA 6009, Australia; sam.salman@uwa.edu.au; 3 Medical School, The University of Western Australia, Perth, WA 6009, Australia; britta.regli-vonungern@uwa.edu.au; 4 Perioperative Medicine Team, Perioperative Care Program, Telethon Kids Institute, Perth, WA 6009, Australia; 5 Department of Anaesthesia and Pain Medicine, Perth Children’s Hospital, Perth, WA 6009, Australia

**Keywords:** flucloxacillin, Eudragit EPO, palmitic acid, taste-masked microparticles, mixture design, paediatric formulation

## Abstract

Flucloxacillin is prescribed to treat skin infections but its highly bitter taste is poorly tolerated in children. This work describes the application of the D-optimal mixture experimental design to identify the optimal component ratio of flucloxacillin, Eudragit EPO and palmitic acid to prepare flucloxacillin taste-masked microparticles that would be stable to storage and would inhibit flucloxacillin release in the oral cavity while facilitating the total release of the flucloxacillin load in the lower gastrointestinal tract (GIT). The model predicted ratio was found to be very close to the stoichiometric equimolar component ratio, which supported our hypothesis that the ionic interactions among flucloxacillin, Eudragit EPO and palmitic acid underscore the polyelectrolyte complex formation in the flucloxacillin taste-masked microparticles. The excipient–drug interactions showed protective effects on the microparticle storage stability and minimised flucloxacillin release at 2 min in dissolution medium. These interactions had less influence on flucloxacillin release in the dissolution medium at 60 min. Storage temperature and relative humidity significantly affected the chemical stability of the microparticles. At the preferred storage conditions of ambient temperature under reduced RH of 23%, over 90% of the baseline drug load was retained in the microparticles at 12 months of storage.

## 1. Introduction

Effective formulation strategies for taste-masking medicines containing a bitter drug are critical to make the medicines acceptable to young children. Bitter-tasting antibiotics in particular are a significant barrier to achieving medication compliance in the paediatric population [1], and flucloxacillin sodium (FS) is a well-documented example. FS is prescribed to children for the treatment of staphylococcal skin and soft tissue infections; however, it is extremely challenging to achieve four times a day dosing compliance with the commercial FS oral liquids due to their highly unpalatable taste [2]. We have formulated a promising taste-masked flucloxacillin ternary microparticle (FTM) formulation consisting of FS, Eudragit EPO (EE) and palmitic acid (PA) [3]. The FTM was hypothesised to be a polyelectrolyte complex (PEC) with FS dispersed throughout a solid powder via a stoichiometric complex formation with EE and PA that would minimise FS release and the detection of its bitter taste in the mouth. The mechanisms for the PEC formation are proposed to involve (1) spontaneous mutual interlocking of oppositely charged molecules, an entropy-driven instantaneous process; (2) reorientation of polymer chains to the correct position for reaction, a slow process that takes 1–2 h; and (3) hydrophobic interaction leading to aggregate formation [4,5]. The reactions between FS, EE and PA under ideal conditions would proceed at a stoichiometric ratio. However, the drug molecules might become entrapped within the aggregates and polymer network, or be adsorbed onto intertwined polymer side chains with unpaired ionic groups, and these could result in nonstoichiometric PEC formation [6]. Further investigation was warranted to determine whether the application of an equimolar ratio of FS, EE and PA (calculated based on the 1:1:1 molar ratio of carboxylic acid of FS: tertiary amine of a functional monomer of EE: carboxylic acid of PA) was ideal for the preparation of the FTM to achieve the desired pharmaceutical attributes. 

The aim of this study was to predict, using a mixture design with D-optimal design points, the optimal composition of FS, EE and FA that would yield FTM with the desired pharmaceutical attributes. The fabrication process for the FTM had previously been optimised [3], and the optimised process was used to prepare the FTM for this study. The critical quality attributes of the FTM were predefined to include its effectiveness at taste-masking FS in the oral cavity, its capacity to release the total FS load in the lower GIT and its stability to storage at ambient temperatures. Three defined in vitro markers served as surrogates for the desired attributes of the FTM. Through in vitro dissolution experiments, the effectiveness of taste-masking was ascertained by measuring FS release in 2 min in simulated saliva, on the assumption the FTM upon administration is unlikely to remain in the mouth for more than 2 min. FDA acceptance criteria for an oral immediate release dosage form containing a water-soluble drug requires the release of at least 80% of the drug load in 30 min in the dissolution medium [7]. The dissolution behaviour of a solid dispersion is however significantly impacted by the formulation design, in particular the physicochemical properties of the drug and excipients [8,9]. The FTM contained significant amounts of PA, a lipophilic agent that could potentially retard particle wetting and FS dissolution. Thus, to determine whether the FTM was able to release FS in vivo, the in vitro dissolution experiments were conducted to measure the cumulative FS released from the FTM at 60 min rather than 30 min. Storage stability was assessed by measuring the residual drug content in the FTM after 6 months of storage at an ambient temperature. 

## 2. Results

### 2.1. Data Analysis for D-Optimal Mixture Experimental Design

A total of 22 FTM samples were evaluated under the D-optimal experimental design. There were subtle differences in the sample texture in that coarse powders with a dry, flaky texture were obtained for FTM with higher EE while fine powders with a fluffy, almost cottony texture were observed for FTM with a higher PA or FS contents. Notwithstanding the initial morphology, all the FTM samples took on the appearance of a fine white powder after they were ground and sieved (212 µm). 

The three measured responses (%R2, %R60 and %Ratio@6M) for the design points are shown in Table 1. The %R2 data fitted into a cubic model (adjusted r2 = 0.87), while the quadratic model was more appropriate for the %R60 and %Ratio@6M data (adjusted r2 = 0.69 and 0.86, respectively). All three models were statistically significant (*p* < 0.05) with insignificant lack of fit (*p* = 0.65, 0.14 and 0.44 for %R2, %R60 and %Ratio@6M, respectively), suggesting the relationship between responses and independent variables did not occur by chance. Summary statistics of the prediction model coefficients and corresponding CI are shown in Table 2.

**Figure 1:**
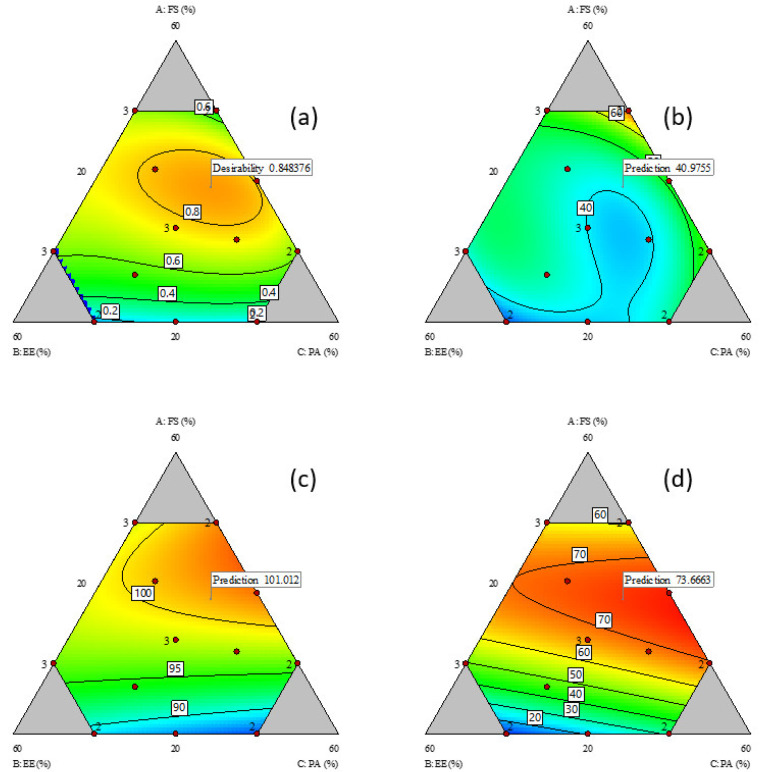
Contour plots of responses; (**a**) desirability plot; (**b**) %R2; (**c**) %R60; and (**d**) %Ratio@6M relative to composition of components; FS = flucloxacillin sodium, EE = Eudragit EPO and PA = palmitic acid. Red dots represent actual design points, and blue contour region has lower response value than red region. Flags represent the model predicted optimal responses to minimise %R2 and maximise %R60 and %Ratio@6M.

The %R2 data was in the range of 29.4% to 74.8% (Table 1). It was increased by higher FS and PA contents (FS, coefficient 110.9 and PA, coefficient 86.5, Table 2), and decreased by drug–excipient interactions, specifically interactions of FS with PA (AC, coefficient −186.6) and FS with EE (AB, coefficient −22.9 and AB(A-B) coefficient −250.7). Conversely, interactions between the excipients, EE and PA, increased the %R2 (BC, coefficient −7.5 and BC(B-C), coefficient 177.1). The contour plot for %R2 (Figure 1b) shows a narrow region (blue) of relatively low %R2 (below 40%) when the FS content in the FTM increased to approximately 40%.

The %R60 data for all the design points indicated that more than 80% of the FS load in the FTM were released into the dissolution medium at 60 min (Table 1). The %R60 was influenced to similar magnitudes by the content of the three components (FS: coefficient 95.3, EE: coefficient 90.3 and PA: coefficient 84.4) (Table 2). At low FS levels, the %R60 increased linearly with FS content (Figure 1c), which is expected as FS has higher water solubility than EE and PA. However, at FS content higher than 30%, the %R60 no longer showed a linear response, indicating that the excipients (EE and PA) had some influence on the %R60. While the interaction terms were significant, the coefficient values were nonetheless smaller than the interaction coefficients for %R2. 

There was large variation (range 9 to 77) in the %Ratio@6M values (Table 1), which suggests that the component composition strongly influenced the FTM storage stability. Interactions between FS and excipients had a protective effect on the FTM chemical stability (FS-EE, coefficient 1462.8, and FS-PA, coefficient 1094.2, Table 2) whereas interactions between the excipients, EE and PA, did not affect the %Ratio@6M. The highest %Ratio@6M value was observed when FS was approximately 40%, with EE and PA combined at 60% (Figure 1d).

**Figure 2:**
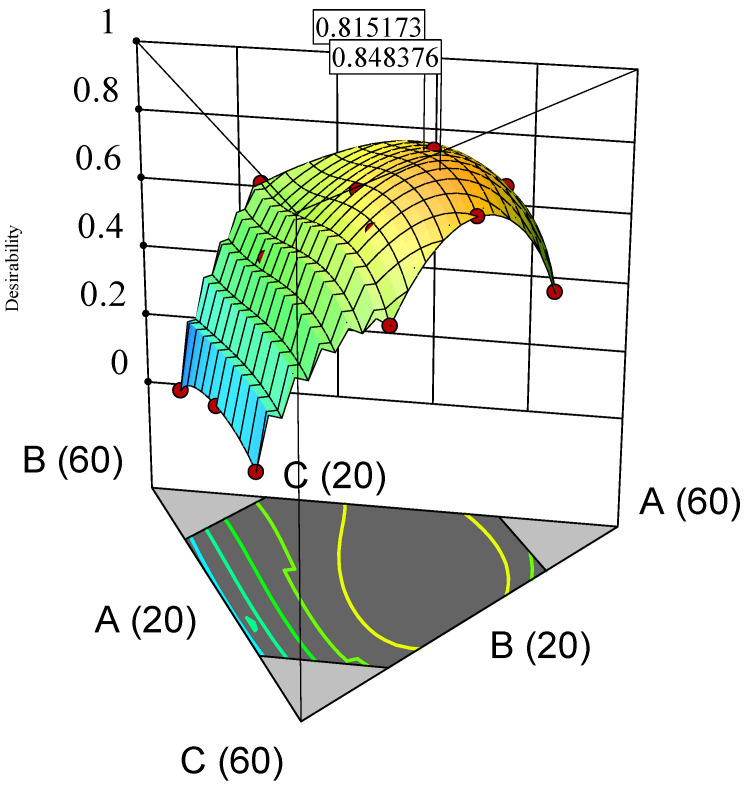
3D surface desirability plot showing a flag at model-predicted optimal component ratio (MCR, desirability index 0.848376) and a flag at equimolar component ratio (ECR, desirability index 0.815173). Vertices labelled as A (60), B (60) and C (60) indicate FS 60%, EE 60% and PA 60%, *w*/*w*/*w*, respectively. Lines labelled as A (20), B (20) and C (20) represent FS 20%, EE 20% and PA 20%, *w*/*w*/*w*, respectively. FS = flucloxacillin sodium, EE = Eudragit EPO and PA = palmitic acid.

A contour plot was also created for the desirability index, which represented the geometric mean of the three transformed responses of %R2, %R60 and %Ratio@6M. The region of desirable component ratio, defined as the area where the desirability index was higher than 0.8, was located within a narrow circle of the contour desirability plot (Figure 1a). The MCR was one that had the desirability index at maximum value, i.e., where %R2 was minimised, and %R60 and %Ratio@6M were maximised. The maximum desirability index of 0.85 corresponded to the weight ratio of 39% FS, 26% EE and 35% PA, which was taken to be the component ratio of the FTM-MCR. The desirable component ratio region also included the point where the component weight ratio was at the equimolar component ratio (FTM-ECR with FS: EE: PA at 42:33:25 *w*/*w*/*w*). The difference in desirability index between the FTM-MCR and FTM-ECR was small (0.85 vs. 0.82) (Figure 2).

### 2.2. DSC Thermograms

**Figure 3:**
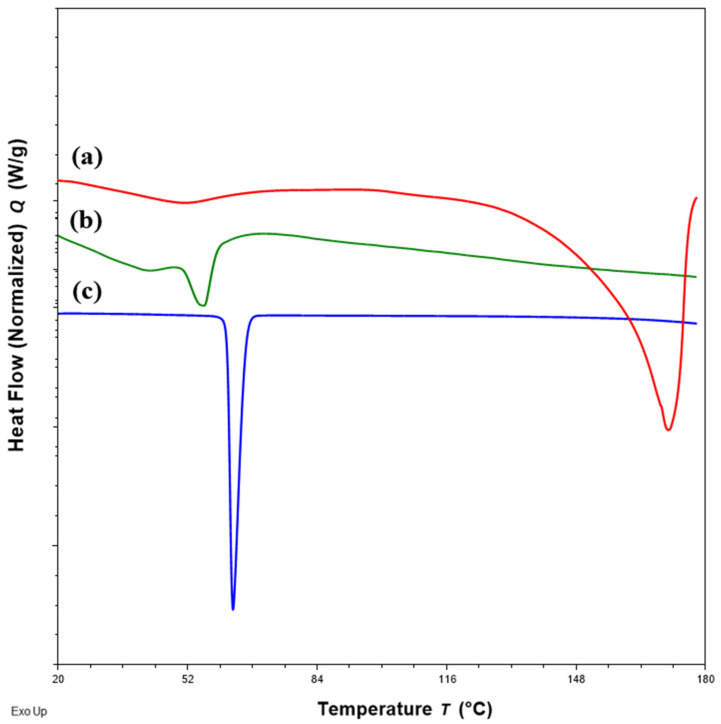
DSC thermograms of (**a**) flucloxacillin sodium (FS, red); (**b**) Eudragit EPO (EE, green); (**c**) palmitic acid (PA, blue) showing the peak temperatures of the endotherm of FS, EE and PA.

**Figure 4:**
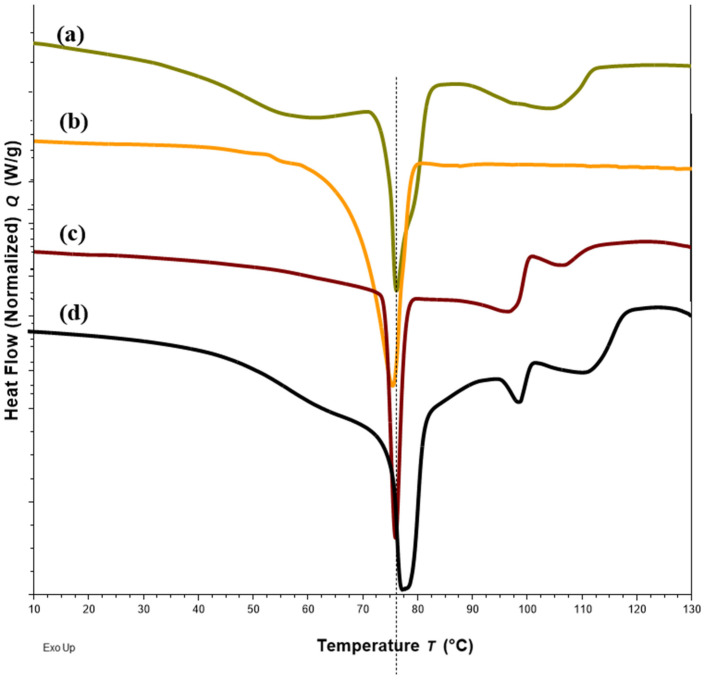
DSC thermograms of Sample 18 with FS-EE-PA at 20:50:30 *w*/*w*/*w* (**a**), Sample 20 with FS-EE-PA at 30:20:50 *w*/*w*/*w* (**b**), Sample 22 with FS-EE-PA at 50:20:30 *w*/*w*/*w* (**c**) and FTM-ECR with FS: EE: PA at 42:33:25 *w*/*w*/*w* (**d**). FTM-ECR = fabricated at equimolar component ratio, FS = flucloxacillin sodium, EE = Eudragit EPO and PA = palmitic acid.

DSC thermograms of FTM samples at the three extremities of the model design points, Sample 18 (20%FS:50%EE:30%PA), Sample 20 (40%FS:20%EE:40%PA) and Sample 22 (50%FS:20%EE:30%PA), were analysed with reference to the DSC thermograms of the individual components (Figure 3) and FTM-ECR (Figure 4). Samples 18, 20 and 22 exhibited the typical prominent endotherm seen in FTM-ECR, but with slightly lower peak temperatures of around 76 °C (Figure 4). Sample 18, which had a high EE content, also showed a Tg at around 50 °C suggestive of the presence of unreacted EE. Sample 20, which had a high PA ratio, showed a single broad endotherm over 60–80 °C that suggests overlapping endotherms attributable to PA and FTM. Of the three samples, only Sample 22, which had high FS and a composition closest to FTM-ECR, showed the two distinct endotherms seen with the FTM-ECR at 76 °C and 96 °C, respectively, and the additional exotherms beyond 110 °C that are likely to be associated with FS degradation [3]. Similar to FTM-ECR, Samples 18, 20 and 22 did not exhibit the FS melting peak at 170 °C, indicating a transformation of FS from a crystalline to amorphous structure when incorporated into the FTM.

### 2.3. Particle Size and Morphology

**Figure 5:**
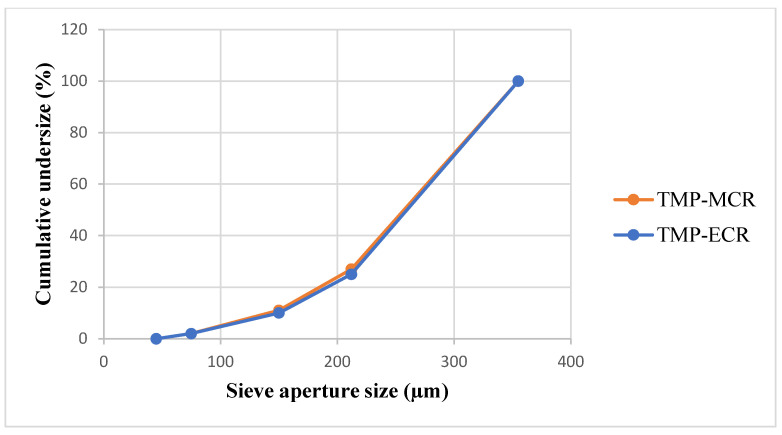
Particle size distribution for FTM-MCR (20 g, n = 1) and FTM-ECR (20 g, n = 1) samples. FTM samples were milled using a pestle and mortar and the particle size was analysed by the sieving method.

FTM-MCR and FTM-ECR showed similar appearances although FTM-ECR was coarser in texture. Milling of the samples in a mortar followed by size analysis by sieving showed that over 70% by weight of FTM-MCR and FTM-ECR comprised of particles in the size range of −355 + 212 µm (Figure 5), and the two samples had comparable mass distribution in the size ranges <45 µm, −75 + 45 µm, −150 + 75 µm, −212 + 150 and −355 + 212 µm. The number of particles in each size range was estimated by dividing the mass of FTM retained on the sieve by (sieve aperture diameter)^3^, and the highest number of particles was found in the size range of −75 + 45 µm for both FTM samples. 

**Figure 6:**
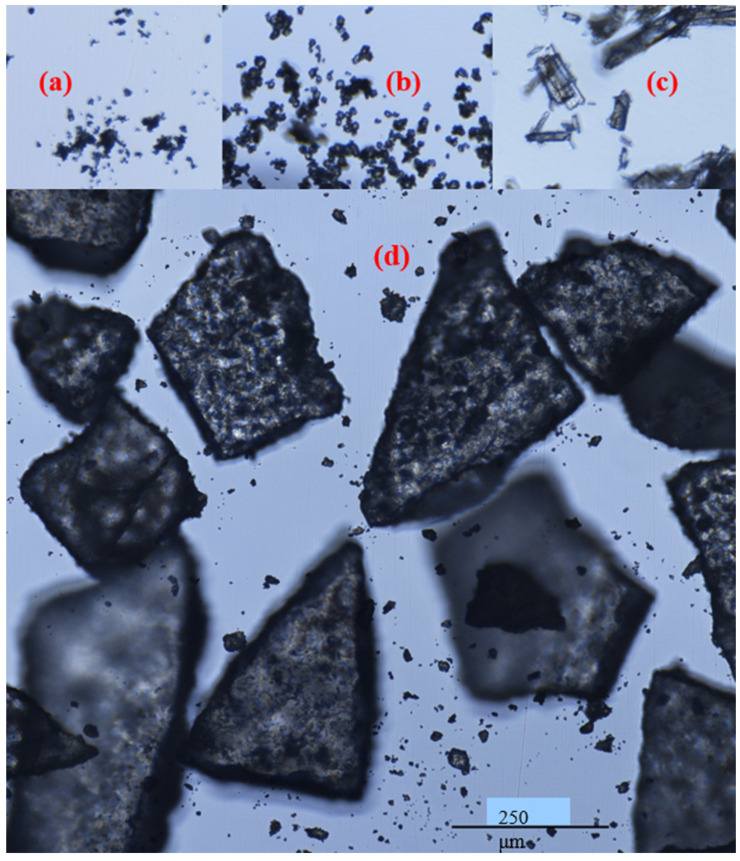
Appearance of (**a**) flucloxacillin sodium; (**b**) Eudragit EPO; (**c**) palmitic acid; (**d**) milled FTM-ECR under a light microscope, magnification 400×; Nikon Eclipse Ti2.

FTM-ECR after size reduction appeared as irregular plates of low sphericity under the microscope (Figure 6d), and this was also observed for FTM-MCR after size reduction. In contrast, FS appeared as aggregates of particles of a variety of shapes under the microscope (Figure 6a) while EE were thin transparent plates (Figure 6b) and PA were rod-shape particles (Figure 6c). Moreover, the particles in FS, EE and PA were all much smaller in size compared to those in FTM-MCR and FTM-ECR. Some FTMs had lengths longer than 355 µm, which suggests that they might have passed through the 355-µm sieve through their shorter length. FTMs with a diameter less than 45 µm were also observed, even though they did not yield a detectable mass when analysed by sieving (Figure 6d).

### 2.4. Storage Stability

#### 2.4.1. Drug Content

**Figure 7:**
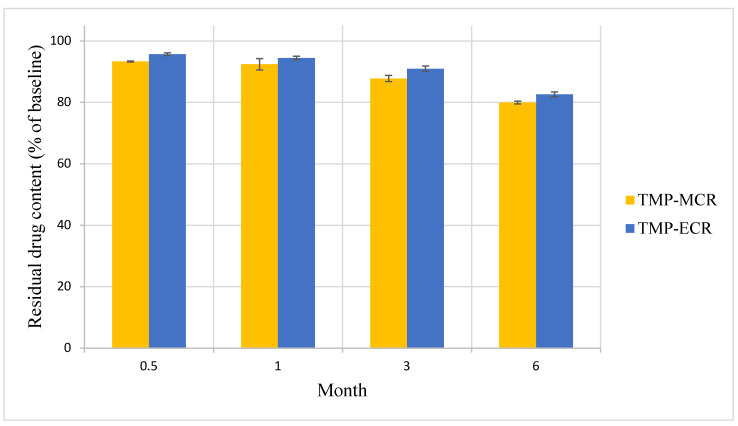
Stability of FTM-MCR and FTM-ECR samples stored at ambient temperature and relative humidity of 32 to 79%. Residual flucloxacillin sodium content in the FTM was determined by HPLC analysis at the specified duration of storage and expressed as a percent of baseline content. Data represent mean ± SD (n = 3).

**Figure 8:**
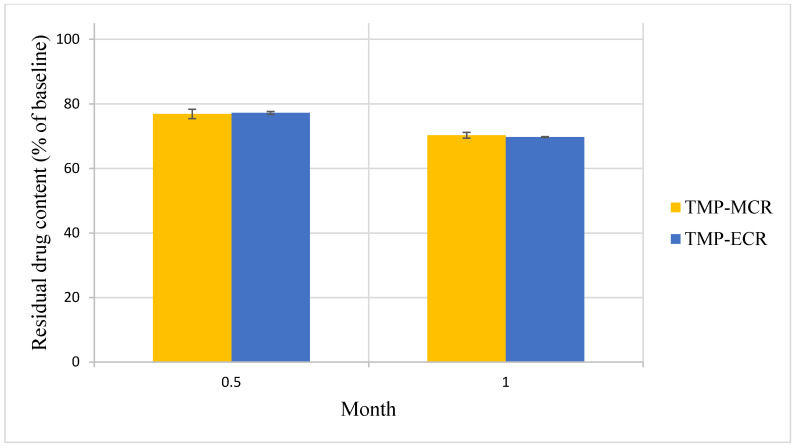
Stability of FTM-MCR and FTM-ECR samples stored at 40 °C and relative humidity of 44 to 52%. Residual flucloxacillin sodium content in the FTM was determined by HPLC analysis at the specified duration of storage and expressed as a percent of baseline content. Data represent mean ± SD (n = 3).

There was no statistically significant difference in the Day 0 baseline drug content between the two FTM samples (*p* = 0.041, n = 3); the FS content was 39.3 ± 1.5 and 39.9 ± 0.4% *w*/*w*, respectively, for FTM-MCR and FTM-ECR. The two FTM samples also showed comparable chemical stability profiles. At 6 months of storage at ambient conditions, the residual FS content as a percent of baseline for the FTM-MCR was 79.9 ± 1.2%, while that for the FTM-ECR was not statistically different at 82.6 ± 1.9% (*p* = 0.057, 95% CI) (Figure 7). When stored at the accelerated stability evaluation condition of 40 °C, both FTM samples were more unstable, but still showing comparable rates of drug loss of more than 20% after 14 days and more than 30% after 1 month (Figure 8). 

**Figure 9:**
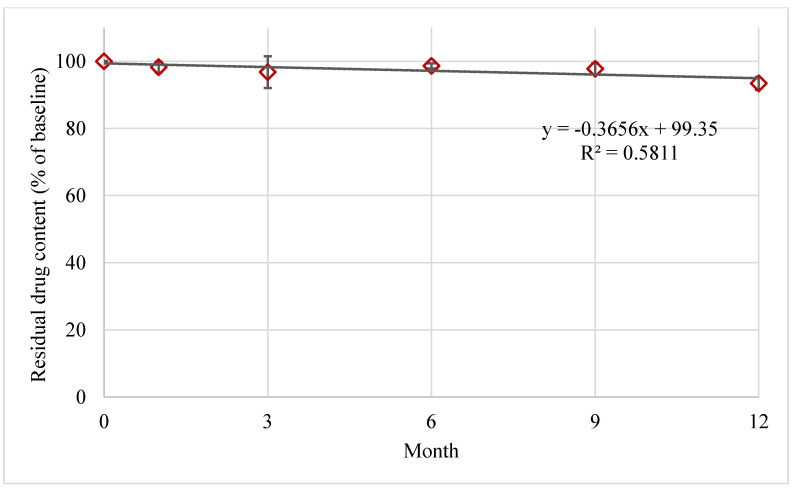
Stability of FTM-ECR samples stored at ambient temperature under reduced relative humidity of 23%. Residual flucloxacillin sodium content in the FTM was determined by HPLC analysis at the specified duration of storage and expressed as a percent of baseline content. Data represent mean ± SD (n = 3). The black solid line represents a linear regression line.

FTM-ECR was further investigated for its storage stability at ambient temperatures under reduced RH of 23%. At 6 months of storage at an ambient temperature, the mean residual FS content in FTM-ECR stored at 23% RH was higher than 96% whereas the mean residual FS content in FTM-ECR stored at atmospheric RH (32 to 79%) had fallen below 90% (Table 3). Even on the prolongation of storage time to 12 months, the FTM-ECR samples remained chemically stable (>90% mean residual FS content) at the reduced RH conditions (Figure 9). 

The effects of RH on the storage stability of FTM-ECR was further studied by changing the RH during storage. A transfer of FTM-ECR after 9 months of storage at ambient temperatures under reduced RH (residual FS content 97.7 ± 1.6%) to storage at ambient temperatures and RH (57 to 79%) for 7 days led to a loss of 3.5% drug content (residual FS content 94.2 ± 3.8%). Prolonging the storage at ambient RH to 14 days did not change the mean FS content (95.3%); however, there was a larger variation (±9.1%) in the residual FS content in the triplicate samples.

#### 2.4.2. DSC

**Figure 10:**
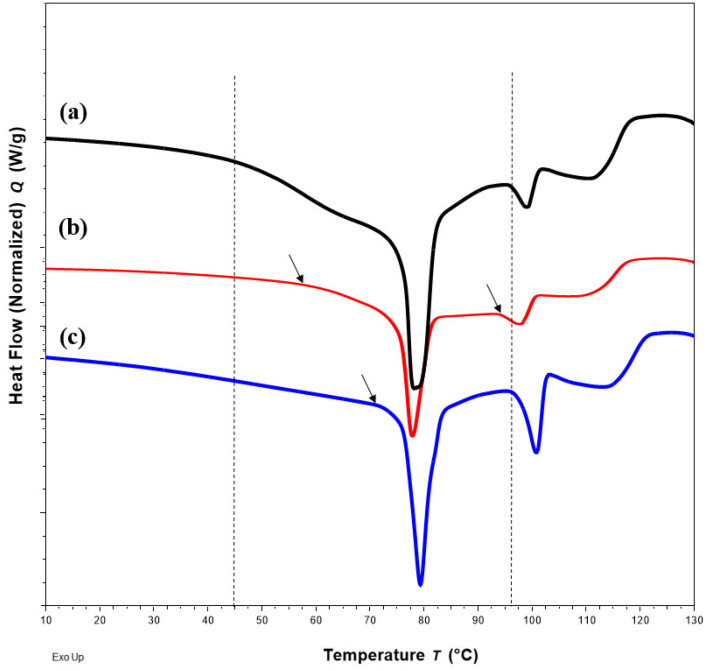
DSC thermograms for (**a**) FTM-ECR samples at Day 0; (**b**) after 3 months storage at ambient temperature and relative humidity (RH) of 57 to 79%; (**c**) at ambient temperature under reduced RH of 23%. Black dash lines show the onset of endotherms for the FTM-ECR at Day 0, with arrows indicating a shift in onset for endotherms after 3 months storage.

**Figure 11:**
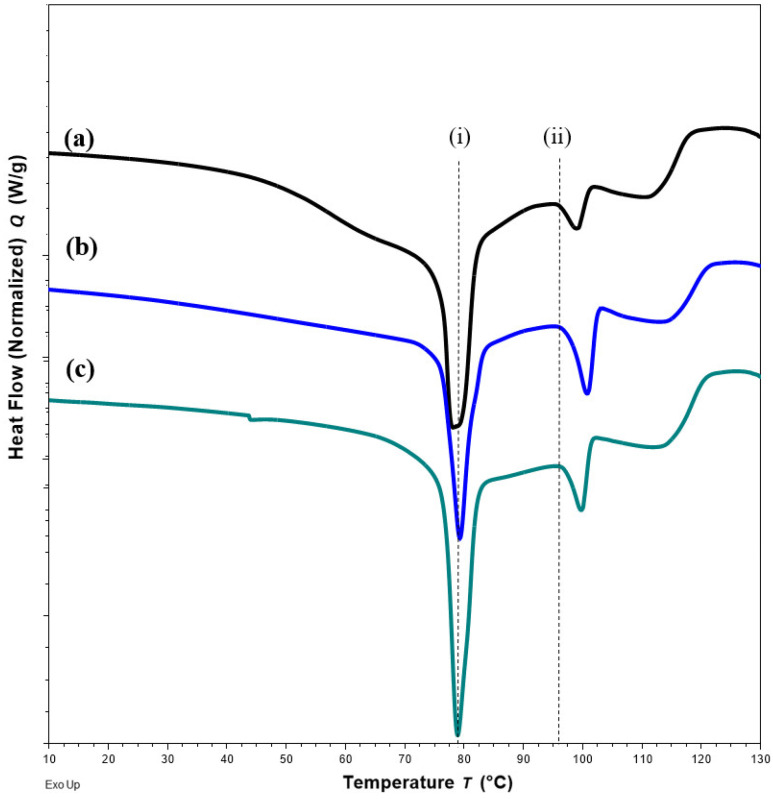
DSC thermograms for FTM-ECR samples (**a**) at Day 0; (**b**) after 3 months; (**c**) after 4 months of storage under relative humidity of 23%. All samples were stored at ambient temperature. Black dash lines show no shift in the peak (i) and the onset (ii) of the specified endotherms.

**Figure 12:**
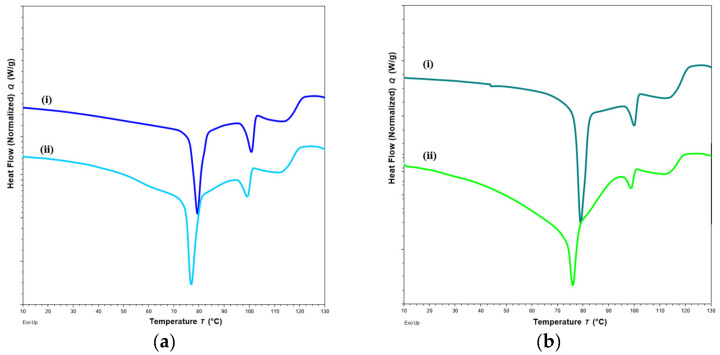
DSC thermograms of FTM-ECR samples after (**a**) 3 months; (**b**) 4 months of storage at relative humidity (RH) of 23%, and before (i) and after (ii) transfer to storage for 14 days at RH of 57 to 79%. All samples were stored at ambient temperature.

Changes in DSC thermograms were observed for FTM-ECR after storage at different conditions. FTM-ECR stored for 3 months at RH 57 to 79% showed a downward shift of 3 °C for the onset temperature of the endotherm at 100 °C (Figure 10b). Conversely, FTM-ECR stored at reduced RH of 23% showed an increase in the peak area but no apparent change in the onset temperature for this endotherm (Figure 10c). FTM-ECR stored for 3 months also showed a sharper endotherm at 78 °C (Figure 10b), with samples stored at reduced RH showing a more prominent sharpening of this peak than those stored at higher RH (Figure 10c). The onset of this endotherm also shifted from about 45 °C to higher temperatures of 58 °C and 71 °C for samples stored at RH 57 to 79% and RH 23%, respectively. FTM-ECR stored under reduced RH for 3 months and longer for 4 months showed similar DSC thermograms (Figure 11), suggesting that the storage-related events were completed by 3 months of storage under reduced RH. Transferring the FTM-ECR after 3 months storage at reduced RH to storage for 14 days at RH 57 to 79% caused the DSC endotherm at 78 °C to broaden and to span across 50 °C to 83 °C (Figure 12a). A similar broadening of this endotherm was observed for FTM-ECR stored for 4 months at reduced RH and transferred to 14 days storage at RH 57 to 79% (Figure 12b), with the endotherm now observed from 40 °C to 83 °C.

### 2.5. In Vitro Drug Dissolution Profile

**Figure 13:**
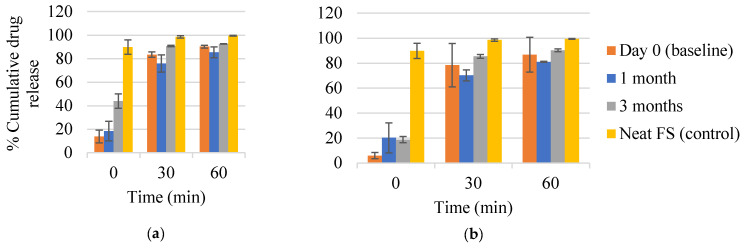
In vitro dissolution profiles of (**a**) FTM-MCR; (**b**) FTM-ECR samples on Day 0 and after 1 month and 3 months storage at relative humidity of 57 to 79%. Dissolution was performed over 60 min in 90 mL of PBS (pH 6.8) with Tween 80 0.1% *w*/*v*, at 37 °C and magnetic stirring at 100 rpm. Data are presented as mean ± SD (n = 3) of cumulative drug release expressed as a percent of drug load in the sample. FS = flucloxacillin sodium (control).

FTM-MCR and FTM-ECR at Day 0 showed the release of at least 80% of the respective drug loads within 60 min of incubation in simulated intestinal fluid (Figure 13a,b). Drug released at time 0 was more than two-fold higher for FTM-MCR (13.8 ± 5.4%) than for FTM-ECR (*p* = 0.0301, 6.0 ± 2.4%), with both samples showing significantly retarded initial drug release compared with neat FS (89.9 ± 6.1%). Storage for 3 months at ambient temperature and RH 57 to 79% increased the initial drug release from both FTM samples, with a greater change observed for FTM-MCR (*p* = 0.003, Figure 13a,b). Despite this increase, the stored FTM samples still showed a lower initial drug release than neat FS. Storage for 3 months at ambient temperature and RH 57 to 79% did not affect the cumulative drug release at 60 min, with both FTM samples releasing well above 80% of their respective drug loads (FTM-MCR, 92.6 ± 0.2%; FTM-ECR, 90.3 ± 1.1%). 

**Figure 14:**
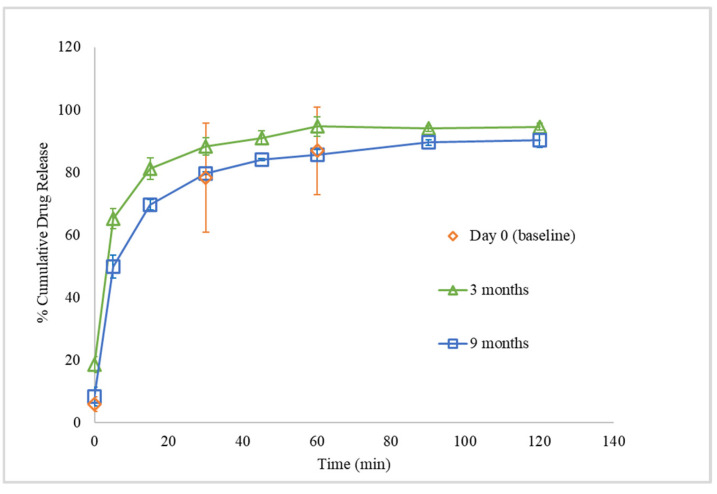
In vitro dissolution profiles of FTM-ECR samples on Day 0 and after storage for 3 months and 9 months at ambient temperature and relative humidity of 23%. Dissolution was performed over 120 min in 90 mL of PBS (pH 6.8) with Tween 80 0.1% *w*/*v*, at 37 °C and magnetic stirring at 100 rpm. Data are presented as mean ± SD (n = 3) of cumulative drug release expressed as a percent of drug load in the sample.

Storage under reduced RH also affected the in vitro drug dissolution profile of FTM-ECR. While the triplicate Day 0 samples showed highly variable drug release data from each other, the triplicate samples stored at reduced RH for 3 months and 9 months showed less variable drug release profiles (Figure 14). Additionally, at 3 months, FTM-ECR stored under reduced RH showed 4.4% higher mean cumulative drug release at 60 min (94.7 ± 3.1%) compared to FTM-ECR stored at atmospheric RH (*p* = 0.0897). A prolongation of storage at reduced RH from 3 months to 9 months caused the FTM-ECR to exhibit 10% lower drug release at all sampling time points and lower cumulative drug release at 60 min (85.6 ± 1.8%), although the drug release did reach 90.3 ± 2.3% at the longer dissolution time of 120 min (Figure 14).

### 2.6. Swelling of FTM in Dissolution Medium

**Figure 15:**
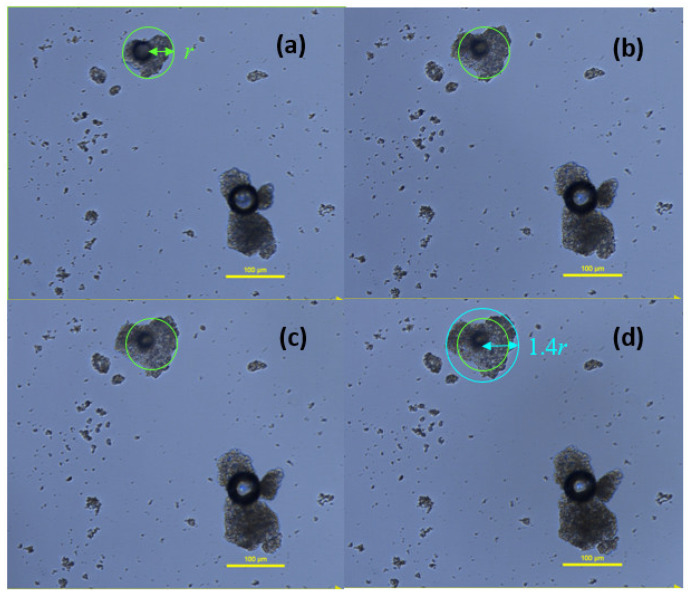
Images of FTM-ECR particles as a function of incubation time in phosphate buffered saline, pH 6.8, with 0.1% *w/v* Tween 80. Images were taken at (**a**) 2 min; (**b**) 15 min; (**c**) 30 min; (**d**) 60 min under a light microscope, magnification 400×; Nikon Eclipse Ti2. Green circle with radius r indicates diameter of particle at time 0 on the assumption the particle is a sphere, and blue circle with radius equivalent to 1.4 r shows final diameter of particle at 60 min.

FTM-ECR particles suspended in three drops of dissolution medium were observed under a microscope to increase in diameter over time, suggesting swelling behaviour (Figure 15). However, the particle shape did not change, which might indicate uniform swelling of the particle matrix. By drawing a circle that enclosed the largest width of the FTM-ECR particle at time 0 and again at 60 min of incubation (Figure 15), it was determined that the particle radius increased 1.4-fold and the particle volume by 2.7 fold, assuming sphericity.

## 3. Discussion

In this study, the DoE approach was successfully applied to identify the composition of the FTM-MCR to meet the collective goal of minimising %R2, and maximising %R60 and %Ratio@6M. The compositional ranges within which the three components were constrained were well-defined in that they enabled the identification of the FTM-MCR within the design space. The data also supported our hypothesis of stoichiometric reactions between FS, EE and PA which point to the FTM-MCR having a component ratio near to the theoretical calculated ECR. At this ratio, optimal ionic interactions between FS, EE and PA existed to increase FS stability as well as to reduce FS release at 2 min in the simulated saliva medium. This finding suggests that the FTM-MCR was a polyelectrolyte complex of FS, EE and PA that formed a close to stoichiometric equimolar ratio.

The fabrication process utilised a solvent evaporation method where FS, EE and PA were all solubilised prior to PEC formation. Reactions between the components were therefore expected to be entropy driven, and therefore instantaneous, involving molecules at their thermodynamically preferred orientations. Nonetheless, not all of the model design points contained equimolar component compositions, and some might end up containing unreacted components, as was exemplified by Sample 18, which showed a glass transition at 50 °C that points to the existence of unreacted EE in the sample. The unreacted components were likely to be the cause of the FTM samples having different textures within the model design space. 

The %R2 response, a surrogate marker for drug release in saliva, ranged from 29.4% to 74.8% for the 22 FTM samples in the model design space. These values are relatively high for a taste-masked product; however, they were obtained at a dissolution time of 2 min, which is longer than the typical time taken by a patient to swallow the FTM. The volume of dissolution medium applied (90 mL for 6.8 mg FS) was also greater than the physiological production of saliva, at 1 mL/min [10], in the oral cavity. There is no official in vitro dissolution test for the human oral cavity, and the %R2 response was designed to be a simple screening tool that was unlikely to equate to the true amount of FS release from the FTM in the human oral cavity. The %R2 response was nevertheless helpful in assessing initial drug release from the FTM, and the relative values of %R2 could therefore be reasonably applied to predict the optimal component ratio for the FTM. 

FS is highly thermolabile and prone to hydrolysis in the presence of moisture due to the chemical reactivity of the fused thiazolidine ring and highly strained β lactam ring in its chemical structure [11]. Flucloxacillin is a penicillin analogue, and its primary degradation product, penicillenic acid, is likely responsible for flucloxacillin’s degradation [3]. This study shows that FS interactions with EE and PA in the FTM provided some protective effects on the FTM’s storage stability. This is helpful as the inherent FS properties make it highly challenging to develop a child-appropriate taste-masked FS medicinal product. 

The model further indicated that FS interactions with EE and PA lowered %R2, but the %R60 response was influenced to a lesser extent, suggesting that prolonged incubation in the dissolution medium diminished the effects of these interactions on FS release from the FTM. Both PA and EE are not soluble in the dissolution medium. Incorporation of the hydrophobic PA, in particular, could affect the wetting of FTMs in the aqueous medium. EE is insoluble at pH 6.8 but the hydrophilic polymer could be hydrated, and this would account for the swelling of the FTMs in the dissolution medium. For FS to be released from the FTM, the FS load must be dissociated from ionic interactions with EE followed by diffusion of the dissociated FS molecules through the FTM matrix into the dissolution medium. The hydration of EE leading to a swelling polymer network could sterically hinder FS release initially; however, the significant dilution effect when the FTM was added to the dissolution medium would facilitate FS dissociation once the FTM was adequately wetted. Hence, the PA and EE levels in the FTM affected the FS release at earlier time points (%R2) but once the FTM was adequately wetted with prolonged incubation in the dissolution medium, the PA and EE levels exerted less effects on the FS release, i.e., the %R60 response. This is helpful as the PA and EE levels could be manipulated to a certain extent to reduce the %R2 response without affecting the %R60 response for the FTM. 

FTM-MCR and FTM-ECR could be considered to exhibit comparable storage stability based on residual FS content analysis alone. However, FTM-ECR based on the %R2 responses might release less FS load initially upon contact with an aqueous medium, which would be helpful for taste masking. The properties of FTM-ECR are also more robust, as its composition is based on a component molar ratio, which is readily adjusted for different batches of starting materials. By comparison, the composition of FTM-MCR is based on a specific component weight ratio determined by the DoE in this study, and its properties could be sensitive to inter-batch variation in the functional group density of the excipient source materials. For these reasons, FTM-ECR would be a more favourable formulation than FTM-MCR. 

FTM-ECR was highly sensitive to temperature and RH during storage, with lower RH found favourable for the storage at an ambient temperature. In general, amorphous solids are stable when stored at temperatures at least 50 °C below their Tg [12]. At higher storage temperatures, in particular temperatures higher than the Tg, the higher molecular mobility coupled with the accompanying reorientation of molecules can increase the instability of the amorphous solid. FTM-ECR at Day 0 exhibited an endothermic transition with the onset at about 45 °C, while unreacted EE in the FTM had Tg of about 50 °C. The application of the 50 °C rule would suggest that the FTM was stable only if stored at temperatures below the refrigerated temperature (2–8 °C). The storage stability data indeed suggest that the FTM-ECR was unstable when stored at ambient conditions, where the recorded temperature over the 12-month storage duration, which included summer and winter seasons, had ranged from 18 °C to 27 °C, and the RH had ranged from 32 to 79% (average 54%). Storage temperature might, however, not be as critical as the RH during storage because the FTM-ECR sample was found to be stable for at least 12 months when stored at ambient temperatures when the RH was reduced to 23%. The storage stability of FTM-ECR could therefore be affected by the extent of moisture sorption from the environment. Absorbed moisture could facilitate molecular mobility [13] and destabilise the hydrogen bond between FS and EE [14] in the FTM. 

DSC analysis also showed a narrowing of the endotherm at 78 °C that was indicative of reduced molecular mobility in the FTM-ECR stored at lower RH. The higher molecular mobility at Day 0 for the FTM-ECR could be attributed to residual solvent. Even though Day 0 was defined as the day when the samples registered no further weight change on drying, the analytical balance (precision ±0.1 mg) might not be able to detect the low amounts of residual ethanol potentially still present in the samples. The boiling point for ethanol is 78 °C, which would overlap with the FTM endotherm around 78 °C. The narrowing of this peak following storage, whether at ambient conditions or reduced RH, could therefore reflect the loss of residual ethanol from the FTM. When the FTM-ECR samples stored under low RH were transferred to storage at ambient conditions, the reduced molecular mobility was reversed, reflecting the effect of moisture sorption on the FTM, even though FS content analysis showed only low levels of drug loss following the change in storage condition (*p* = 0.0982). Another observable change in the DSC thermograms for samples which showed greater storage instability was the lowering in onset temperature of the endotherm at about 100 °C, which again was suggestive of a higher molecular mobility of the hygroscopic FTM samples. 

During storage, amorphous solid dispersions are known to show aging effects, wherein the molecules assume orientation of a thermodynamically lower energy status and the free volume is reduced [15,16]. Recrystallisation and phase separation can also occur. As the free volume decreases [17], the matrix structure becomes more compact, which may retard the water permeation and drug dissolution rate [18]. This phenomenon was observed with the FTM-ECR samples stored for 3 months at ambient conditions, as well as FTM-ECR samples stored for 9 months under reduced RH. Nonetheless, the aging of the FTM samples was not significant enough to reduce the drug release to below 80% at the sampling time point of 60 min. DSC is recognised by the regulatory authorities as an acceptable method for discerning between amorphous and crystalline structures, making it an appropriate choice for this study. Future studies could include X-ray powder diffraction data to provide unequivocal proof of the amorphous content of the FTM powder.

The FTM-ECR sample was stable for at least 12 months stored at ambient temperatures and reduced RH of 23%. The stability of the FTM sample stored under reduced RH has not been studied at other temperatures. Based on the current data, it may be proposed that the FTM-ECR sample is best stored in a vacuum-sealed container at an ambient temperature. Translation to practice suggests that once the vacuum seal is breached, FTM stability could be compromised. FS prescribed as an antibiotic usually has a treatment course lasting 5 days, in some cases extending to 14 days. This study showed small but highly variable drug losses in FTM-ECR, which retained a mean residual FS content above 90% of the baseline when it was transferred from storage at reduced RH to storage for up to 14 days at ambient conditions. Thus, a conservative recommended packing size for the FTM-ECR would be for 7 days treatment.

This study has some limitations. The DoE model, while providing a useful approach to screen factors to achieve the desired pharmaceutical attributes for the FTM, may not be generalisable to other experimental conditions. In particular, the particles in the FTM were size-reduced to less than 212 µm before sampling for the assessment of responses and storage stability. Furthermore, EE as a polymer has inter-batch differences in properties. As an example, the potentiometric titration of the EE used in this study showed that it contained 3.605 mmol/g of the critical dimethylaminoethyl functional group. However, commercial EE has been reported to contain variable levels of this amino group, ranging from 2.86 to 4.28 mmol/g [19], and one group has reported an even higher level of 5.6 mmol/g [20]. Thus, FTM-MCR could have different component ratios depending on the property of the EE used. Our study mitigated this limitation by proposing FTM-ECR as a model supported component ratio alternative. 

Another limitation is the dissolution medium used. FS by itself is not soluble in simulated gastric pH, yet the FTM contained EE, which is soluble in gastric pH but not intestinal pH. Preliminary studies have shown that approximately 20% of an FS load was soluble in the presence of EE at gastric pH due to supersaturation. Flucloxacillin is less prone to degradation in gastric acid due to the electron withdrawing effect of the fluoro group in the isoxazole heterocycle [11]. However, a two-step in vitro dissolution test comprising incubation in 0.1 M HCl followed by incubation in PBS indicated that the FS that had dissolved in the simulated gastric fluid under the influence of EE was not fully detectable in the simulated intestinal fluid. Thus, an improved design of the dissolution experiment may be required to better simulate the in vivo bioavailability of FTM.

Further study is required to formulate a stable oral age-appropriate dosage form for young patients using the FTM microparticles. One strategy is to employ a dry formulation such as an orally dispersible tablet packaged in a vacuum-sealed closure system. Another alternative could be in situ mixing of the FTM with an oral gel that could delay the FS diffusion from the TMP to the taste receptors and aid rapid swallowability of the TMP. Considering that children aged from 6 months can overcome the extrusion reflex to swallow thick semi-solids [21], the in situ TMP-gel formulation may be administered to patients as young as 6 months old.

Besides FS, there are other drugs in the form of sodium salts that are highly bitter, including dicloxacillin sodium, sodium docusate, diclofenac sodium and naproxen sodium. Based on the proposed reaction mechanisms, the methodology for the FTM may be extended beyond FS to the taste-masking of these bitter drugs as sodium salt with EE and PA. Future study is required to generalise this approach, as laid out in our two-part manuscripts, to mask the bitter taste of a drug presented as a sodium or other cation salt form, as well as to further develop the FTM formulation into a child-appropriate oral dosage form.

## 4. Materials and Methods

### 4.1. Materials

The following materials were used: flucloxacillin sodium (FS) monohydrate (Aspen Pharmacare Australia Pty Ltd., St Leonards, Australia), Eudragit^®^ EPO (Evonik Industries, Frankfurt, Germany), palmitic acid (ThermoFisher Scientific, Malaga, Australia), ethanol (Chem-Supply Pty Ltd., Gillman, Australia), acetone (RCI Labscan, Bangkok, Thailand) and potassium dihydrogen phosphate (Ajax Chemicals, Sydney, Australia). Unless specified, all chemicals and reagents were of analytical grade. 

### 4.2. Methods

#### 4.2.1. Experimental Design

The D-optimal experimental design was generated (Design-Expert 12 software, Stat-Ease Inc., Minneapolis, MN, USA) with the independent variable defined as a mixture component ratio, expressed as percent weight fraction of FS, EE and PA (%FS, %EE and %PA, respectively). The upper and lower constraints for the component weight fractions were determined from a preliminary study and consideration of clinical drug dosing, and were applied at FS level: 20–50%, EE level: 20–50% and PA level: 20–50%. The measured responses were % drug release at 2 min (%R2) and % drug release at 60 min (%R60) in the dissolution medium, and residual drug content after 6 months of storage at ambient temperature measured as a ratio to the baseline drug content at Day 0 (%Ratio@6M). Within the design space, the desired ratio of components for the FTM would be one that exhibits minimum %R2, maximum %R60 and maximum %Ratio@6M.

A total of 22 runs were computer generated to include the six required model points, three additional model points, three additional centre points, five lack of fit points and five replicate points (Table 4). All runs were randomised to reduce hidden sources of variation.

Statistical analysis was performed using the Design-Expert ^®^ (version 12) software. A polynomial model was fitted for each response using multivariate regression analysis. The best fit model was selected based on adjusted and predicted r square values, the model significance *p* value (*p* < 0.05) and lack of fit p value (*p* > 0.05). After the models were selected, the significance of model terms was evaluated by backward elimination. The selected final models were evaluated using diagnostic plots, such as the residual plots, Cook’s distance plot and normality plot of residuals.

To determine the optimum component ratio that met the collective goal for the three responses, i.e., minimum %R2, maximum %R60 and maximum %Ratio@6M, a single new variable, the desirability index, was created to reflect all three responses simultaneously. The desirability value for each measured response was ranked 0 to 1, with 1 being most desirable, and the geometric mean of the three transformed responses was assigned to a new desirability index. The model predicted optimal component ratio (MCR) was one that had the desirability index at maximum value.

#### 4.2.2. FTM Preparation

FTMs were prepared at the specified component ratio (Table 4) by mixing an FS solution and a binary PA + EE solution. The total weight of components (FS, EE and PA) in all samples was 0.5 g. In brief, FS was dissolved in 1 mL of 1:4 *v*/*v* ethanol: acetone, and PA together with EE were dissolved in 2 mL of ethanol. The FS solution was added to the binary PA + EE solution under magnetic agitation for 5 min at 350 rpm, and the resultant suspension was decanted onto baking paper to dry overnight at an ambient temperature to yield the FTM. To minimise particle size bias on the %R2 and %R60 responses and to facilitate uniform sampling, the FTMs after drying were milled in a mortar and sieved (aperture size 212 µm, Glenammer Laboratory Test Sieves, Ayr, Scotland, UK) before transferring into 10 mL clear capped glass vials for storage at an ambient temperature (18–27 °C). The sample weight was monitored until no further weight change occurred before the samples were further analysed and the day when no further weight change was recorded was regarded as Day 0 for the baseline assessment of the FTMs.

The manufacture process was scaled up to prepare batch sizes of 20 g for the FTM fabricated at the model-predicted optimal component ratio (FTM-MCR) and the FTM fabricated at the equimolar component ratio (FTM-ECR, equivalent to weight ratio of FS:EE:PA at 42:33:25 *w*/*w*/*w*). The FTMs were milled in a mortar and characterised for size distribution. The samples on the sieves and receiver pan were then pooled and transferred into a 10 mL clear capped glass vial for storage at an ambient temperature (18–27 °C). the sample weight was monitored until no further weight change occurred, and the day when no further weight change was recorded was regarded as Day 0 for the baseline assessment of the FTMs.

#### 4.2.3. Particle Size Reduction and Analysis

The FTM-MCR and FTM-ECR samples were assessed for size distribution using sieve analysis. Samples of 20 g were passed through a series of sieves in descending aperture diameter (355, 212, 150, 75 and 45 µm) by vibrating the sieves for 15 min. Any powder remaining on the top sieve (355 µm) was collected for milling using a mortar and pestle, and the sieving procedure was repeated until all powder passed through the top sieve. The weights of powder on the remaining sieves (212, 150, 75 and 45 µm) and collecting pan were recorded for size analysis.

#### 4.2.4. Drug Content

FTM (15 mg) was dissolved by sonication for 30 min with 1.5 mL of ethanol, and 100-µL samples were aliquoted into 10 mL glass test tubes. Each sample was mixed by vortex for 1 min with 4 mL of diluent (25 mL acetonitrile, 75 mL of 20 mM potassium phosphate buffer (pH 5.0), with 0.75 g KCl added to reduce ionic interactions among components). The diluted samples after filtration (0.2 μm × 13 mm nylon syringe filter, Agilent Technologies Australia, Mulgrave, Australia) were analysed for drug content by high performance liquid chromatography (HPLC). The HPLC was calibrated with standard solutions prepared by diluting a stock solution (10 mg of FS, 8 mg of EE and 6 mg of PA in 1.5 mL of ethanol) with the diluent to give concentrations in the range of 0.03–0.16 mg/mL.

The drug content of the FTM samples was quantified using a validated HPLC method. HPLC analysis was performed on an integrated Agilent 1260 Infinity II binary LC system (Agilent Technologies Australia, Mulgrave, Australia) with an Agilent 1260 Infinity diode-array detector. Chromatographic separation was carried out at 20 °C on a reversed phase C18 column (Hypersil ODS column, 150 × 4.5 mm, 5 µm particle size, Thermo Fisher Scientific, Scoresby, Australia) coupled with a Hypersil Classical C18 Guard Cartridge (5 µm, 10 × 4.6 mm). Isocratic elution was applied with a mobile phase comprising 25:75 *v*/*v* of acetonitrile and potassium phosphate buffer (20 mM, pH 5.0) and flow rate of 1 mL/min. The sample injection volume was 20 µL at an ambient temperature, and the eluent was monitored at 225 nm for FS quantification. Total run time was 20 min. Chromatographic data were collected and processed using the Agilent OpenLAB CDS software (Version 2.4).

#### 4.2.5. Storage Stability

Storage stability was assessed by analysing the residual intact drug remaining in the FTM after storage at specified conditions. Baseline assessment of samples was performed at Day 0 (the day when no further weight change occurred, approximately 7 days after fabrication). A summary of the storage stability study design is given in Table 5.

For all model points in the D-optimal design space, the drug content of the FTM was analysed at Day 0 and 6 months of storage. Analysis was performed on aliquoted samples (200 mg) stored in capped 1 mL clear glass vials in a laboratory cupboard at ambient conditions. The temperature ranged from 18 to 27 °C, while the relative humidity (RH) was 32 to 79% over the storage period (Supco DVTH device, Sealed Unit Parts Co., Inc., Wall Township, NJ, USA). %Ratio@6M was calculated as the percent of drug content at 6 months storage relative to the respective baseline drug content at Day 0. 

FTM-MCR and FTM-ECR samples on Day 0 were aliquoted (2 g) and stored in capped 10 mL clear glass vials each also containing 1 g sachet of silica gel desiccant (Wisesorbent Technology, Marlton, NJ, USA). Drug content assay was performed on triplicate samples at Day 0, after storage for 0.5 and 1 month at 40 ± 5 °C, RH 44 to 52% (UF160 oven, Memmert, Schwabach, Germany), and after storage for 0.5, 1, 3 and 6 months at ambient conditions (temperature 18 to 27 °C, RH 32 to 79%). Additionally, the stability of FTM-ECR was assessed over a period of 12 months stored at an ambient temperature and reduced RH (~23%), which was achieved by placing the FTM vials in a glass desiccator (diameter 300 mm, Pacific laboratory products, Blackburn, VIC, Australia) connected to a central vacuum line. To further evaluate the effects of RH on storage stability, FTM-ECR samples stored for 9 months at an ambient temperature and reduced RH (~23%) were transferred to storage for 14 days at ambient conditions (RH 57 to 79%). The drug content assay was performed for triplicate samples immediately prior to the change in the storage RH, and after 7- and 14-days storage at ambient conditions.

#### 4.2.6. In Vitro Dissolution Profile

The in vitro dissolution method was adapted from the method of Steiner et al. [22] with slight modifications as the British and US pharmacopoeias do not have a dissolution method for FS. Phosphate buffered saline (PBS), pH 6.8 with 0.1% *w*/*v* Tween 80 was used as the dissolution medium. Simulated gastric acid (0.1 M HCl) was the preferred medium to measure drug release in the lower GIT as its acidic pH would facilitate EE dissolution and therefore FTM disintegration; however, it could not be applied in this study because FS was insoluble in 0.1 M HCl. Instead, PBS was used, and Tween 80, a non-ionic surfactant, was added to aid FTM wetting in PBS. Dissolution was performed at 37 °C, with stirring at 100 rpm (radleys TECH magnetic stirrer, In vitro Technologies, Noble Park North, Australia). FTM equivalent to 6.8 mg of FS was weighed into a 100 mL beaker and dissolution was initiated by adding 90 mL of dissolution medium. Neat FS at 6.8 mg stored at the same conditions as the FTM samples was used as control. 

For all model points in the D-optimal design space, the in vitro dissolution experiment was performed at Day 0. Aliquots of 1 mL were withdrawn from the dissolution medium at 2 min and 60 min. To compare the in vitro dissolution profiles of FTM-MCR and FTM-ECR, the in vitro dissolution experiment was performed on triplicate samples on Day 0, and after 1- and 3-months storage at ambient conditions. Aliquots of 1 mL were withdrawn from the dissolution medium at 0, 30 min and 60 min. Sampling at time zero was performed immediately after the dissolution medium was added to the samples, ensuring an accurate representation of the initial conditions. To assess the in vitro dissolution profile of FTM-ECR after storage at ambient temperatures under reduced RH (23%), the dissolution study was performed in triplicates at 0, 3 and 9 months of storage. Aliquots of 1 mL were withdrawn from the dissolution medium at predefined intervals. All withdrawn aliquots were syringe filtered (0.2 μm × 13 mm nylon syringe filter, Agilent Technologies Australia, NSW, Australia) and 20 µL of the filtrate were injected for HPLC analysis. Aliquots withdrawn were immediately replaced with an equal quantity of fresh dissolution medium.

#### 4.2.7. Particle Morphology and Swelling of FTM in Dissolution Medium

The gross morphology of dry FTM particles was observed under a light microscope (Nikon Eclipse Ti2 microscope). FTM behaviour in the dissolution medium was monitored by placing 20–25 FTM particles with 3 drops (~150 µL) of dissolution medium in a well (96-well microplate). The plate was covered to minimise solvent evaporation, and images of the particles observed under the microscope at 2, 15, 30 and 60 min were taken (Nikon DS-Ri2 camera) and processed (NIS-Elements BR Analysis 4.60.00).

#### 4.2.8. Analysis by Differential Scanning Calorimetry (DSC)

To assess for component interactions, DSC analysis was performed on FTM samples at three model design points, Sample 18 (20%FS:50%EE:30%PA), Sample 20 (40%FS:20%EE:40%PA) and Sample 22 (50%FS:20%EE:30%PA). To examine the effects of storage on the FTM-ECR samples, DSC analysis was carried out on Day 0 and after the specified storage conditions. All samples were weighed (2–5 mg) and sealed into aluminium pans (40 µL) for DSC analysis under a nitrogen gas purge (50 mL/min) at heating rates of 10 °C/min over the temperature range of −10 °C to 170 °C (Discovery DSC 25, TA instruments, New Castle, DE, USA, liquid nitrogen quench cooling). An empty pan served as reference. Thermograms were analysed using the Trios software (Version 5.0.0., DE, USA).

## 5. Conclusions

The DoE was successfully applied using a D-optimal mixture design to identify the FTM-MCR in the model design space to achieve the desired pharmaceutical attributes. The model supported the ECR as an alternative ratio predicted based on the functional groups of the three components being the desirable ratio to achieve the desired pharmaceutical attributes of the FTM. The FTM was moisture- and temperature-sensitive. It was able to be stably stored for at least 12 months at ambient temperatures under a reduced RH of 23%. An in vitro dissolution test showed over 80% of the FS load was released at 60 min even after 9 months of storage under the reduced RH. 

## Figures and Tables

**Table 1:** Percent weight fraction of components (independent variables) and the three measured responses obtained from the D-optimal mixture experimental design. %R2 and %R60 represent percentage drug release at 2 min and 60 min, respectively, in PBS-Tween 80 (pH 6.8, 100 rpm at 37 °C), and %Ratio@6M represents residual drug content in FTM after 6 months of storage at an ambient temperature 18 to 27 °C and relative humidity 57 to 79%, expressed as a percent to the baseline drug content at Day 0. FS = flucloxacillin sodium, EE = Eudragit EPO and PA = palmitic acid.

Sample Number	Composition of Components (% *w*/*w*)			Measured Response		
	%FS	%EE	%PA	%R2	%R60	%Ratio@6M
1	33	33	33	35.2	95.2	73.5
2	20	30	50	44.6	84.9	33.7
3	33.	33.	33	41.1	100.7	50.0
4	42	32	27	41.3	92.6	62.1
5	50	30	20	49.3	96.8	62.0
6	50	30	20	59.9	102.4	63.1
7	30	50	20	45.3	99.4	47.8
8	27	42	32	44.7	95.7	46.2
9	20	40	40	38.6	89.1	9.4
10	32	27	42	44.5	100.4	77.2
11	20	50	30	29.4	86.3	10.0
12	30	50	20	43.9	95.9	56.7
13	33	33	33	42.0	103.2	71.5
14	30	50	20	39.6	95.6	53.6
15	50	30	20	53.6	99.3	53.9
16	30	20	50	57.9	93.1	58.9
17	20	30	50	44.9	87.3	37.8
18	20	50	30	36.2	87.5	9.1
19	50	20	30	65.9	104.4	58.6
20	30	20	50	57.9	94.7	73.2
21	40	20	40	48.8	103.5	74.7
22	50	20	30	74.8	100.1	66.6

**Table 2:** Model coefficients and confidence intervals (CI) for the three responses (%R2, %R60 and %Ratio@6M) measured for FTM samples prepared with different compositions of flucloxacillin sodium (A), Eudragit EPO (B) and palmitic acid (C). Data were fitted to the cubic model for %R2 and the quadratic model for %R60 and %Ratio@6M.

	Coefficient Estimate	95% CI
%R2		
A	110.9	91.9–129.9
B	−5.4	−38.6–27.9
C	86.5	66.3–106.7
AB	−22.9	−83.4–37.6
AC	−186.6	−271.8–−101.4
BC	−7.5	−60.7–45.6
AB(A-B)	−250.7	−381.2–−120.2
BC(B-C)	177.1	45.4–308.9
%R60		
A	95.3	87.3–103.4
B	90.2	84.2–96.3
C	84.4	78.3–90.5
AB	26.6	−4.1–57.3
AC	48.5	21.3–75.8
%Ratio@6M		
A	−386.0	−527.9–−244.2
B	−221.5	−313.7–−129.4
C	−45.2	−131.0–40.6
AB	1462.8	1006.2–1919.5
AC	1094.2	689.3–1499.1

**Table 3:** Residual flucloxacillin sodium content (% of baseline level) (n = 3, mean ± SD) in the FTM-ECR samples after 1, 3 and 6 months of storage at ambient temperatures under an atmospheric relative humidity (RH) of 32 to 79% and reduced RH of 23%.

Storage Condition	Duration of Storage (Months)		
	1	3	6
Atmospheric RH	94.5 ± 0.6	91.0.5 ± 0.9	82.6 ± 0.77
Reduced RH	98.2 ± 1.6	96.8 ± 4.8	98.6 ± 0.8

**Table 4:** Percent weight fraction of components (FS, EE and PA) and actual weight of FS, EE and PA employed for the D-optimal mixture design. There was a total of 22 experiments which included 6 required model points, 3 additional model points, 3 additional centre points, 5 lack of fit points, and 5 replicate points. Total weight of components for each model point was 0.5 g. FS = flucloxacillin sodium, EE = Eudragit EPO and PA = palmitic acid.

Sample Number	Percent Weight Fraction of Components			Actual Weight of Components Used		
	%FS	%EE	%PA	FS (g)	EE (g)	PA (g)
1	33	33	33	0.17	0.17	0.17
2	20	30	50	0.10	0.15	0.25
3	33	33	33	0.17	0.17	0.17
4	42	32	27	0.21	0.16	0.13
5	50	30	20	0.25	0.15	0.10
6	50	30	20	0.25	0.15	0.10
7	30	50	20	0.15	0.25	0.10
8	27	42	32	0.13	0.21	0.16
9	20	40	40	0.10	0.20	0.20
10	32	27	42	0.16	0.13	0.21
11	20	50	30	0.10	0.25	0.15
12	30	50	20	0.15	0.25	0.10
13	33	33	33	0.17	0.17	0.17
14	30	50	20	0.15	0.25	0.10
15	50	30	20	0.25	0.15	0.10
16	30	20	50	0.15	0.10	0.25
17	20	30	50	0.10	0.15	0.25
18	20	50	30	0.10	0.25	0.15
19	50	20	30	0.25	0.10	0.15
20	30	20	50	0.15	0.10	0.25
21	40	20	40	0.20	0.10	0.20
22	50	20	30	0.25	0.10	0.15

**Table 5:** A summary of the storage stability study design showing the purpose, storage conditions and samples (n = 3) used for each experiment. FTM: Flucloxacillin Taste-Masked Microparticle, FTM-MCR: fabricated at the model predicted optimal component ratio, FTM-MCR: fabricated at equimolar component ratio.

Samples	Purpose	Storage Condition	Time Points for Drug Content Analysis
FTM of all model points in the D-optimal design space	To measure model response, %Ratio@6M	Ambient temperature, relative humidity (RH) 32 to 79%	0 and 6 months
FTM-MCR and FTM-ECR	Storage stability at ambient conditions	Ambient temperature, RH 32 to 79%	0, 0.5, 1, 3 and 6 months
FTM-MCR and FTM-ECR	Accelerated stability study	40 ± 5 °C, RH 44 to 52%	0, 0.5 and 1 month
FTM-ECR	Storage stability at reduced RH	Ambient temperature, reduced RH (23%)	0, 3, 6, 9 and 12 months
FTM-ECR	Effects of RH on stability	Ambient temperature, reduced RH (23%) for 9 months, then transferred to ambient temperature, RH 57 to 79%	9 months at ambient temperature and reduced RH, and 7 and 14 days at ambient temperature, RH 57 to 79%

## Data Availability

Data is contained within the article.
